# Unraveling polarization: Insights into individual and collective dynamics

**DOI:** 10.1093/pnasnexus/pgae426

**Published:** 2024-10-15

**Authors:** Kati Kish Bar-On, Eugen Dimant, Yphtach Lelkes, David G Rand

**Affiliations:** Science, Technology and Society Program, Massachusetts Institute of Technology, 77 Massachusetts Avenue Cambridge, MA 02139-4307, USA; Center for Social Norms and Behavioral Dynamics, University of Pennsylvania, 3451 Walnut Street Philadelphia, PA 19104, USA; Annenberg School for Communication, University of Pennsylvania, 3451 Walnut Street Philadelphia, PA 19104, USA; Sloan School of Management, Massachusetts Institute of Technology, 77 Massachusetts Avenue Cambridge, MA 02139-4307, USA

**Keywords:** affective polarization, interventions, measurement methods, group behavior, social norms, collective polarization, individual polarization

## Abstract

Polarization poses a critical threat to the stability of nations around the world, as it impacts climate change, populism, democracy, and global health. This perspective examines the conceptual understanding, measurement challenges, and potential interventions for polarization. Our analysis highlights the distinction and interactions between the individual and collective levels of polarization, conceptually, methodologically, and in terms of interventions. We conclude by pointing out future directions for understanding polarization and highlighting the interrelations between polarization and other social phenomena.

## Introduction

There is growing scholarly concern about polarization, driven by various global political dynamics, including the rise of populist movements (1–3). One particularly worrisome form of this phenomenon is affective polarization, which underscores the emotional and identity-driven divides that have become integral to political discourse. Affective polarization threatens citizens’ willingness to compromise, interact, and hold their representatives accountable (4, 5). We argue that the concept is undertheorized, particularly with regard to how it operates across different levels of analysis and the interactions between individual and collective dynamics. This underscores the need for refined measurement tools and advanced theoretical frameworks to deepen our comprehension of how polarization affects, and is affected by, the interplay between individuals and groups (6). Such an understanding is crucial for addressing the complex ways in which polarization shapes societal interactions and political landscapes.

In this perspective piece, we argue that efforts to measure or define polarization should account for the interactions occurring across the individual-collective spectrum. To substantiate and expand on this framework, we explore 3 distinct research dimensions: a conceptual perspective, measurement methodologies, and cross-level interventions. We address 3 interconnected questions: (i) Is polarization primarily a characteristic of individuals or the collective? (ii) Do current measurement methods assess polarization at the individual level or the extent of division within a collective? and (iii) Which interventions are effective at influencing levels of polarization? By focusing on these questions, we aim to deepen the understanding of polarization dynamics and enhance the effectiveness of strategies to mitigate it.

## The complexity of polarization: definitions and dynamics

Polarization can be categorized into different types, including political, affective, social, economic, cultural, geographical, and media. Our focus is primarily on affective polarization, which indicates an emotional aversion and distrust toward political adversaries. Polarization has many meanings, and each meaning has, potentially, unique causes, manifestations, and consequences. It encompasses a range of interconnected phenomena, such as social identity, group dynamics, trust, biases, beliefs, norms, emotions, and misperceptions, yet these are often examined in isolation. While there has been some exploration of the relationships between these phenomena and different types of polarization (5, 7–10), it is equally important to consider the interplay between individual and collective dynamics.

Collective polarization refers to the degree of division or emotional hostility between groups at the societal level. It encompasses how entire communities, parties, or demographic groups become more ideologically or affectively distant from each other, often manifesting in widespread group-based behaviors, emotions, and norms that reinforce divisions. In contrast, individual polarization pertains to the degree to which a single person becomes more ideologically extreme or affectively hostile toward members of opposing groups. It reflects personal changes in beliefs, emotional reactions, identification processes, and behaviors that move toward the poles of an issue or conflict. Both forms of polarization are interconnected: individual polarization contributes to collective polarization, and collective dynamics, in turn, shape individual attitudes and behaviors.

Polarization can also be defined across the process-state spectrum. The term *polarization* may denote either a static, temporal snapshot (the configuration of a population at a given moment) or an evolving process (the dynamics of a population's configuration over time). Certain formal measures of polarization [e.g. (11)] are attributes of distributions of cardinal-valued beliefs assessed at specific moments in time. These measures allow for the comparison of opinion patterns across various issues or among different populations. Thus, when one observes that American political opinion is polarized, it is generally an articulation of a current, unchanging state; nonetheless, the term can also signify the process of becoming more polarized, such as the escalation of emotional polarization amid electoral campaigns. Additionally, certain forms of polarization are inherently dynamic and cannot be captured through simple comparative analyses of groups or temporal snapshots, such as the gradual radicalization of political discourse on social media over time. For instance, during an electoral campaign, individuals’ discussions on social media platforms may begin relatively moderately but grow increasingly extreme as the campaign progresses. This escalation could be driven by echo chambers, group dynamics, or inflammatory rhetoric from political leaders. Measuring polarization at two points in time (e.g. the beginning and end of the campaign) might reveal increased polarization, but such snapshots miss the daily or weekly fluctuations and the underlying social processes, such as how specific events or speeches accelerate the divide or how group norms shift within subcommunities.

The concept of affective polarization is fundamentally centered on emotion (12) and rests on 3 tenets: polarization is influenced by either positive sentiments toward one's group or negative sentiments toward an opposing group, it involves strongly held beliefs, and it is connected to moral identity—a concept closely linked to emotions (13). Emotions are dynamic, changing in strength and duration, and influenced by both individual and group contexts. Stemming from an individual's identification with a group, group emotions can intensify polarization by driving group identity, solidarity within the group, and animosity toward outsiders (14–16).

The interplay between individual beliefs and collective emotions suggests that polarization is not just a matter of individual psychology or differing opinions, but also a state of the collective that is reinforced by political, media, and social structures. It is a feedback loop in which collective incentives for division increase political homogeneity within groups, which, in turn, deepens collective polarization (17–21). The dynamical interplay between intrapersonal dynamics and interpersonal conflicts (22, 23) suggests that affective polarization requires methodologies that analyze, measure, and comprehend it as more than a fixed state.

Therefore, polarization can be understood, measured, and intervened in across 4 dimensions—individual, collective, process, and state. It can be addressed as a characteristic of individuals, a characteristic of the collective, an ongoing process, a current state of affairs, or any combination thereof. For example, we can conceptualize polarization at the individual level moving across the process-state spectrum, or as a process that moves across the individual-collective spectrum. Regardless of which approach to polarization we adopt, it affects and is affected by the other 3 poles. For example, if we address polarization as a trait of the collective, we must consider that it is measured through individuals’ opinions or behavior. Hence, any change in individuals’ polarized views affects the levels of polarization within the collective. Changes in individuals’ levels of polarization derive from their identification processes, their group categorization, how they perceive social norms, and the extent to which they are exposed to social media. All these factors constantly interact and affect each other, and their impact on individuals’ polarization levels might be immediate or can take longer, depending on the individual's personality and cognitive processes. These dynamics can turn polarization into being seen as a process rather than a state and vice versa.

The individual concept of polarization pertains to the emotional and psychological distance an individual perceives toward members and supporters of the opposing political party. This dimension is characterized by increased hostility, avoidance of cross-party interactions, a tendency to rely on partisan media, and the perception of political opponents as morally inferior. Conversely, the collective concept of polarization refers to the aggregate level of affective polarization within a society, reflecting the broader emotional and psychological division between political groups. This collective dimension manifests as societal divisions along partisan lines, heightened political tension, and significant challenges in achieving bipartisan cooperation.

We argue that both the individual and the collective concepts of polarization impact and influence each other, as they are in constant interaction with other 2-dimensional elements such as norms, emotions, and identity. Norms are societal rules that shape cultures, but they are also elements that can be internalized and become goals in themselves (24–26). Our attitudes toward social norms are shaped by social identification processes, affecting our group identity and national identity, which are both essential for understanding the evolution of polarized views in individuals and groups (27, 28). Emotions affect our levels of identification with the group, as well as our feelings of commitment toward social norms. As such, norms, identity, and emotions not only operate on both the individual and collective levels, but they also interact and affect each other (see Table 1).

**Table 1.** Glossary.

**Affective polarization** refers to the growing emotional divide between supporters of different political parties. At the individual level, it intensifies emotional biases, reinforces partisan identities, skews perceptions, and modifies social behaviors. At the collective level, it disrupts social cohesion, deepens political divides, and diminishes trust in institutions. **Emotions** are conscious or unconscious mental reactions subjectively experienced as strong feelings usually directed toward a specific object. At the individual level, emotions drive motivation and affect cognitive processes, such as attention and memory. At the collective level, they foster group identity, solidarity, and collective action, and impact group response to collective threat. **Norms** are informal rules and standards that guide and constrain social behavior within a group or society. Norms influence individual-level actions through internalization, conformity, and identity, while at the collective level they promote cohesion, mutual reinforcement, and social regulation. **(Social) Identity** is the part of an individual's self-concept derived from perceived membership in a relevant social group. At the individual level, it shapes self-concept, emotional attachment, and motivation. At the collective level, it fosters group cohesion and solidarity, shapes cultural and social norms, and can lead to intergroup bias and conflict.

Individual polarization focuses on personal emotional and behavioral responses, but these responses are also influenced by elements on the collective level, such as group identity and group emotions. Collective polarization encompasses the societal impact and group dynamics arising from widespread affective polarization, yet these group dynamics are shaped by individual-level elements, such as social identity and subjective emotions. To properly understand and accurately define polarization, it is essential to integrate both individualistic and collective perspectives.

Having established the conceptual foundation of individual and collective polarization, we now turn to the practical implications of this framework. In the following section, we explore how these dimensions can be effectively measured, ensuring that interventions are grounded in robust empirical data.

## Measuring polarization across the individual-collective divide

Measuring polarization, a challenging endeavor in itself, becomes even more complex when considering how various methods of measurement address the individual-collective spectrum. Survey-based methods for studying political beliefs and affective polarization are widely used but come with significant drawbacks. One issue is that people often do not answer these surveys sincerely (29). Some might choose extreme positions to show loyalty to a political party or a social group, while others might express opinions on policies they haven't actually thought about, resulting in what is known as “pseudo-attitudes.” To address these limitations, researchers often turn to actual behaviors. For example, analyzing voting patterns is a common approach. Another approach is studying online interactions, in which behavioral polarization is measured by looking at echo chambers, selective exposure to partisan content, or patterns of engagement with partisan figures. Some researchers advocate for using both survey-based and behavior-based approaches to get a more comprehensive picture of affective polarization. Survey data help understand how people feel, while behavioral data show how those feelings translate into actions.

Behavior-based methods offer a more nuanced understanding of affective polarization by focusing on individuals’ actions and decisions. These methods include trust and cooperation experiments, economic decision-making tasks, and analyzing online behaviors, voting patterns, and political contributions. For instance, social media activity, such as engagement within echo chambers, can highlight how polarization influences everyday interactions. Behavioral methods also include tracking political donations, voting behavior, and social network formation to measure how people separate themselves from outgroup members. Additionally, individuals’ choices in media consumption and even their relationships can reflect deep affective polarization. These methods have the advantage of capturing implicit biases and offer insight into polarization's impact on everyday actions. However, interpreting behaviors can be context dependent, and motivations behind behaviors may be influenced by factors other than polarization (such as social identity, cognitive biases, and media exposure).

Survey-based and behavior-based methods primarily measure polarization at the individual level, as they focus on understanding personal ideological stances and the psychological underpinnings of polarization. While these tools offer valuable insights at the individual level, whether their aggregation adequately captures collective manifestations of polarization remains an open question. For instance, it is unclear how individual-level methods, such as survey responses, are affected by the mutual interactions within the individual-collective spectrum. Bullock and Lenz (29) start their paper by examining what constitutes a belief and what drives people to provide incorrect answers. Factors such as group identity, party affiliation, and pro-party heuristics often influence individuals’ responses to opinion surveys, as well as their levels of knowledge and confidence (30, 31). This description highlights several interacting elements from both sides of the spectrum: at the individual level, there are beliefs, knowledge, and confidence levels; at the collective level, there are group identity, party affiliation, and pro-party heuristics. These elements interact and influence each other, continuously moving back and forth across the individual-collective divide. For instance, group identification processes can prompt individuals to alter their beliefs, which can subsequently affect their knowledge and confidence levels. However, surveys measure opinions, beliefs, and biases at the individual level. How, then, does the aggregation of individual beliefs reflect the level of collective polarization?

The recognition that individual-level methods may have limitations in accurately capturing affective polarization has motivated some scholars to turn toward behavioral indicators [e.g. (32–35)]. These methods, which primarily assess trust, altruism, and cooperativeness, offer a novel lens to gauge partisan bias. Whether this bias is a result of partisanship of the partner, per se, versus other constructs that are strongly correlated with a partner's partisanship (e.g. their race or education) is an open question. At the forefront of this development are new validated measurement techniques that create the opportunity to measure important facets of polarization, including the ability to capture pluralistic societal views and norms (36–38). These techniques enable researchers to measure polarization at both the individual level (such as the distribution of norm-related beliefs) and the collective level (including the tightness and looseness of norms) simultaneously, providing a more accurate assessment of polarization.

If we assume that the aggregation of individual-level beliefs accurately captures collective polarization, we must also require statistical tools that account for collective-level processes and dynamics, such as network analysis, geographic analysis, and content analysis. For instance, network analysis methods investigate how polarization develops within social networks by focusing on the formation of epistemic communities. These methods analyze the collective behavior of groups and how their network structures contribute to polarization, rather than concentrate on individual attitudes. Geographic analysis methods map polarization across various regions, examining how political attitudes cluster geographically. They emphasize collective patterns of polarization, demonstrating how regional dynamics and community identities contribute to national political divides. Content analysis methods explore how media content reflects and influences collective political and social dynamics. These methods examine the language, framing, and narratives used in various media sources to understand how polarization is presented and sustained at the societal level.

In line with the conceptual observation in the previous section (The complexity of polarization: definitions and dynamics), we argue that combining measurement methods at both the individual and collective levels is crucial for comprehensively understanding polarization. For example, individual-level measurements of partisan bias can inform collective interventions by identifying key emotional triggers that, when addressed through public discourse reforms, reduce group-level polarization. By integrating methods from both levels, researchers can better understand the interplay between personal experiences and societal phenomena, allowing for more effective interventions and policies to address the root causes and effects of polarization. Possible future experiments that combine measurement methods across both levels could include (i) analyzing individual social media interactions alongside network structures, (ii) studying individual voter attitudes in conjunction with geographic patterns of political behavior, (iii) linking media framing analysis with interpersonal communication studies, (iv) examining public opinion alongside legislative behavior, and (v) integrating cultural activity analysis with political identity studies. Each experiment combines individual-level data with collective-level structures, allowing researchers to see how individual actions contribute to collective outcomes. Accordingly, this holistic approach can shed light on (i) the role of personal behaviors in creating political echo chambers, (ii) the evolution and transformation of regional polarization dynamics, (iii) the impact of media on collective polarization through individual conversations, (iv) the feedback loops between societal attitudes and legislative polarization, and (v) the ways in which cultural factors either exacerbate or alleviate polarization.

Another critical observation derived from the previous section (The complexity of polarization: definitions and dynamics) is that both individual-level and collective-level measurement methods lack an explicit focus on the interactions between and within the process-state spectrum. This oversight limits the understanding of how dynamic processes of polarization (such as changes in attitudes and behaviors) relate to the more static states (such as entrenched ideological positions and societal divides). One of the foundational early studies on polarization (39) viewed ideological polarization as a concept that could be both a state and a process: polarization as a state refers to the degree to which opinions on an issue are opposed relative to a theoretical maximum, and polarization as a process refers to the increase in such opposition over time. Although some studies [e.g. (40)] have recognized DiMaggio's observation, they have argued that determining whether a distribution is polarized is generally a matter of judgment and have not specified which perspective they adopt. This ambiguity makes polarization appear as an even more elusive concept, one that shifts across the process-state spectrum for reasons that are unclear or unaccounted for.

We aim to emphasize that when social scientists measure polarization, they often concentrate on individual-level polarization yet present their findings as relevant to the collective level without adequately explaining the transition or identifying the collective-level measurements employed. For instance, in a recent study, Wojcieszak and Garrett (41) used several individual-level measurement techniques (such as surveys, priming, and media selection) to evaluate the impact of national identity priming on affective polarization. Their findings indicate that at the individual level polarization is influenced by personal attitudes and media choices shaped by identity priming. Collectively, these individual actions coalesce into broader societal patterns of behavior, thereby influencing public discourse and contributing to societal polarization. However, the transition from individual-level methods to collective-level conclusions is not sufficiently clarified, leaving the transformation of individual actions into collective behaviors ambiguous. A conceptual framework, such as the one outlined in the previous section (The complexity of polarization: definitions and dynamics), could have assisted the authors in more effectively situating their findings across the individual-collective spectrum, identifying gaps, and refining their measurement methods for more precise and impactful interventions. By applying this framework, authors can illustrate how the priming of national identity at the individual level contributes to broader collective polarization. This provides a clearer theoretical grounding for understanding how individual biases can escalate into widespread societal divisions. Furthermore, such a conceptual framework can clarify the dynamics of selective media exposure observed in the study by mapping these processes onto the process-state spectrum of polarization. This mapping demonstrates how individual media choices (process) can lead to stable polarized states within society (state), elucidating the complex interplay between personal and collective dimensions of polarization.

In sum, the measurement problem that we want to emphasize revolves around the complexity of polarization as both an individual and collective phenomenon. Measurement methods often fail to clarify what exactly they measure: are they assessing individual levels of polarization or the degree of division within a group, collective, or society? How do measurements of individual polarization levels translate into collective polarization levels? Is collective polarization merely the aggregate of individual polarization levels? We propose that to properly measure polarization, researchers should (i) clearly state whether they are measuring individual-level or collective-level polarization; (ii) aim to incorporate at least 2 measurement methods, one from each level, where feasible, to allow for comparison and validation of results; and (iii) address the interactions between the two levels in their discussion. In the following section we discuss how targeted interventions can address and mitigate the effects of polarization, drawing on the insights gained from our conceptual and measurement frameworks.

## Toward cross-level interventions in polarization research

At the heart of polarization research are assumptions about its deleterious effect on various social and political outcomes. Consequently, quite a bit of research (and money) is devoted to understanding its causes and identify interventions. Interventions to reduce polarization aim to foster understanding, reduce prejudice, and promote dialogue between opposing groups, and largely focus on individual-level solutions.

While these interventions are often successful in lab settings and can change perceptions about the other side or strengthen real or imagined relations with the outparty (34, 42–45), they typically do not address deeper issues related to the collective level, such as democratic norms, group behaviors, and political structures. For instance, while interventions that correct stereotypes of the other side are sometimes effective, they are easily undone in a competitive political information environment (46), in which stereotypes are reset. This example indicates that addressing affective polarization likely necessitates more complex institutional reforms on the collective level, rather than solely relying on behavioral interventions on the individual level. It may be that interventions at the individual level could be more effective when reinforced by interventions at the collective level.

Interventions operate on several levels: (i) the individual level, targeting beliefs, views, and perceptions; (ii) the interpersonal level, affecting interactions between people and their groups; and (iii) the collective level, focusing on reforming political systems, social media influences, and public discourse norms. These levels are not mutually exclusive, and there may be other levels or sublevels (e.g. the collective level includes interventions on groups, institutions, collectives, and random aggregates of people—each requiring a distinct kind of intervention). More importantly, these levels constantly interact, meaning that interventions at one level can impact other levels. For example, an intervention that changes an individual's belief can affect their interactions with people in their ingroups and outgroups. If this individual is a group influencer, this change can affect the entire group's beliefs, potentially triggering a chain reaction of belief changes across their social environment. Conversely, an intervention to change polarizing rhetoric through changes in public discourse norms can shape dialogue norms in smaller groups. If these changes persist, such norms can become part of the group's social identity and eventually be internalized by group members.

**Fig. 1.**
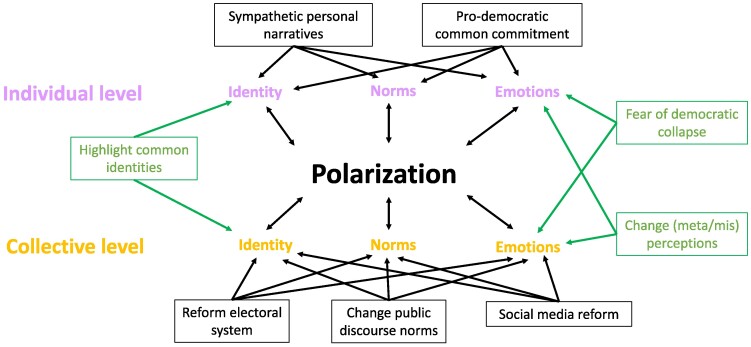
Interventions within and across levels. Polarization is represented here as a continuous process occupying both individual and collective levels. Identity, norms, and emotions are 3 central elements affecting polarization, operating on both levels. Arrows indicate which intervention affects which element and level. Most interventions target either the individual level or the collective level (for example: sympathetic personal narratives, pro-democratic common commitment, reform electoral system, change public discourse norms, and social media reform). We propose that interventions operating on both levels (for example: highlight common identities, fear of democratic collapse, and change meta/mis perceptions) or directly impacting elements on both levels are more likely to succeed in reducing polarization.

We propose that the most effective interventions should incorporate elements that function across all levels, as illustrated in Figure 1. Building upon the conceptual framework outlined previously (The complexity of polarization: definitions and dynamics), Figure 1 presents interventions that target key elements such as norms, emotions, and identity across both the individual and collective levels. It visually demonstrates how interventions can be tailored to operate at the individual level, the collective level, or simultaneously across both. The figure suggests that while traditional interventions often focus on a single level, those addressing both levels are more successful in reducing polarization. For instance, interventions that modify individual emotions while concurrently targeting group emotions can bridge the individual-collective divide, offering more comprehensive solutions to reduce polarization. This approach leverages the interconnectedness of norms, identity, and emotions, as emphasized previously (The complexity of polarization: definitions and dynamics), to design interventions that consider the mutual impact of these elements at both levels. Together, the conceptual framework and Figure 1 illustrate that effective interventions must recognize the complex interplay between individual behaviors and collective societal dynamics. They emphasize that polarization should be understood not merely as an individual issue or a societal problem, but rather as a phenomenon shaped by the interaction between both levels.

The idea here is to use the individual-collective polarization scale to map possible interventions, and derive new intervention ideas from combining existing and new interventions on each level. This way, we can create cross-level interventions that target both individual and collective levels at once, thereby simultaneously affecting both spectrums of polarization. For example, the figure outlines 3 cross-level interventions, with 2 influencing emotions at both the individual and collective levels and 1 impacting identity at both levels. Fear of democratic collapse not only targets individual emotions, but also affects shared values, ideas, and beliefs within a group. At the individual level, framing this fear as a shared, nonpartisan concern can potentially moderate extreme views. By highlighting the common values and shared interests in preserving democratic institutions, individuals from across the political spectrum might feel a unified commitment to the broader democratic process, rather than to partisan success. At the collective level, fear of democratic collapse could foster a sense of shared fate and collective responsibility. When groups recognize that the threat of collapse is a common enemy, it may catalyze collaboration across divides. Targeting both levels could alleviate feelings of insecurity and threat from “the other side,” thereby changing misperception about the “outgroup.” By changing misperceptions, interventions can soften the emotional triggers that drive polarization at both levels. Individuals may feel less threatened by outgroups, and collective emotions of outrage or fear can be diffused. Over time, this can lead to more moderate discourse, increased openness to dialogue, and a willingness to engage in collaborative problem solving.

Another cross-level approach involves community dialogues and media campaigns. These initiatives encourage critical thinking and empathy through storytelling on the individual level and facilitate local dialogue sessions among diverse groups. By partnering with media organizations, these dialogues can be broadcast to promote positive discourse across the collective level. An additional cross-level intervention is creating educational programs that combine civic engagement with school curriculums. These programs incorporate media literacy and bias recognition modules on the individual level and organize student-led projects requiring cross-political collaboration. Partnering with educational institutions can ensure that these initiatives become integral parts of the curriculum, fostering long-term impact on the collective level as well.

Workplace diversity initiatives, public policy forums with deliberative polling, and social media platforms promoting civil discourse are other examples (47–49). On the individual level, these interventions provide training on unconscious bias, encourage collaboration on social responsibility projects, and integrate features to highlight diverse viewpoints and fact-check misinformation. By collaborating with corporations and tech companies, these initiatives can shape organizational culture and online discourse norms on the collective level, ultimately fostering a more inclusive and understanding society.

Clearly, such cross-level interventions are harder to design and test, as it might take a while before we can measure their impact across both levels. They might also be harder to implement, given the many interacting elements, some of which may be harder to control than others. Nonetheless, we believe that this approach might be the most fruitful way forward, even if it requires designing more complex experiments. Polarization is a complex concept that leads to many disagreements among scientists regarding the appropriate definitions, measurement methods, and effective interventions. We believe that this complexity and dissatisfaction are intrinsic to understanding complex social reality; the variety and heterogeneity of interacting elements and levels of operation mirror the genuine complexity found in social phenomena such as polarization. Hence, there is no easy solution—if we aim to reduce polarization, it must be a communal, interdisciplinary effort incorporating conceptual analyses, theoretical overviews, and experimental interventions across multiple levels.

## Concluding remarks

Polarization has emerged as a significant factor influencing the fabric of democracy and societal cohesion around the world. This phenomenon is both influenced by and influences the interactions between individuals including their social identities, values, emotions, and beliefs, as well as larger collectives, including social groups, institutions, communities, and political parties. Understanding polarization requires a multilevel analysis that spans from conceptual frameworks to measurement techniques and the development of interventions. We emphasize the importance of considering the interactions across the individual-collective spectrum, which are essential for a comprehensive exploration of polarization at various levels of analysis.

As we navigate the nuances of polarization and its implications, our discussion extends to the potential pathways for addressing and mitigating its divisive effects on society. To better understand these effects, we must first understand the ways in which differences in opinion shape emotions, beliefs, and actions. Such differences can affect individual's private emotions and actions, but they can also affect emotions and actions at the group level. This distinction is often overlooked when measuring and defining polarization, and our goal is to bring the individual-collective dynamics to the front.

In this perspective piece, we proposed understanding polarization as a concept that encompasses the interplay between the individual-collective and process-state divides, as well as the dynamics between the elements within those divides. As such, we raise several related questions on 3 levels: conceptual, methodological (measurement), and experimental (interventions). On a conceptual level, we ask whether polarization is a property of the collective or the individual and highlight the need for addressing both dimensions and their interactions to get a detailed picture of the phenomenon. On a methodological level, we ask how measuring individuals’ degrees of polarization translates into collective degrees of polarization, and emphasize the advantages of assessing polarization at both levels concurrently. On an experimental level, we examine interventions across the individual-collective spectrum and suggest that cross-level interventions, which address polarization at both the individual and collective levels simultaneously, hold the most promise for effectively mitigating polarization.

Understanding polarization as a 2-fold phenomenon that operates at both the individual and collective levels provides significant benefits to behavioral sciences by offering a comprehensive framework for analyzing the complexities of polarized societies. This perspective allows researchers to explore how individual attitudes and behaviors aggregate into societal patterns, creating a feedback loop that sustains and intensifies polarization. For example, in political communication, this dual-level approach enables scholars to examine how media narratives interact with individual media consumption patterns to shape public opinion. In public policy and governance, understanding polarization at both levels helps policymakers design more effective strategies that address the root causes and societal implications of polarization, fostering social cohesion and bipartisanship. In social psychology, exploring individual identity processes alongside collective social identities enables psychologists to develop interventions that reduce intergroup conflict and promote empathy.

To conclude, we advocate for an approach that views polarization as a continuous interaction between individuals and collectives, which affects and is affected by both levels, and should be measured and intervened with across these divides. Such an understanding is vital for devising strategies that can mitigate political and social division and promote a more cohesive society. Doing so would enable both researchers and practitioners to better capture the richness of our societies and develop more effective solutions and policy implications.

## Data Availability

The manuscript has no associated data.

## Preprint

This manuscript was previously posted as a preprint: https://papers.ssrn.com/sol3/papers.cfm?abstract_id=4784194.
